# Observations on Calculous Disorders

**Published:** 1831

**Authors:** 


**Observations on Calculous Disorders.** By *B. C. Brodie*, Esq. F.R.S., as delivered by him in his Surgical Lectures. Medical Gazette, Nos. 174, et sequent.

Of the importance of an accurate knowledge of the diseases of the urinary organs no practical surgeon nor physician can entertain a doubt. It is true that many difficulties stand in the way of arriving at that knowledge, but the majority are not insuperable, and consequently become a stimulus rather than an obstacle to the success of ingenious and enterprising men. In most diseases, at all events of those organs or tissues which are veiled from sight, we deduce the probable alterations in their structure by more cognizable alterations of their functions. By observing the latter at the bed-side of the sick, and noting the morbid changes of structure presented to our view after death, we couple and connect the one with the other, we mark, compare, and reason, we bring into play the inductive method of philosophizing, and rescue the practice of medicine from much of the obscurity thrown around it, by the empiricism or dogmatism of former ages. It is in this manner, and in this alone, that we can attain to any certain and positive information in medical science. It is by linking symptoms with organic alterations that we ascertain the nature of disease. This is not the whole, to be sure, but it is the foundation on which all that is solid is to be reared, and without it all systems and theories were built but to decay.

In studying the diseases of the urinary organs it is necessary to proceed on precisely the same plan as in the investigation of other organic maladies. We must explore the functions of the parts, both natural and deranged, and mark the corresponding structural alterations; on this is to be erected the only scientific system of diagnosis and cure. Accordingly we find that the ablest and most philosophical inquirers of modern times have proceeded in this manner. Wollaston, Brande, Marcet, Prout, Bazelius, and many others, have devoted their attention to the healthy and unhealthy conditions of the urine. With the exception of Dr. Prout, few have attempted in a philosophic manner to connect those conditions of the urinary secretion with particular states of the system in general or of the secreting organs in particular. On Dr. Prout's investigations we can hardly pass too high an encomium; they evince a degree of patient enquiry, and of sound induction, which place him high amongst the scientific men of the day. We shall presently see that Mr. Brodie will bear comparison with any, even the best of those whose names we have recorded.

The chemical examination of the urinary secretion presents peculiar difficulties to the analyst. If the other fluids of the body exhibit variations they are rather of degree than of kind, rather consisting in alterations of the relative quantities of ingredients, than of the composition of those ingredients themselves. With the urine it is otherwise. It varies in its characters in health, it varies ten times more in disease. To catch and fix each fleeting feature, would be as vain and as unprofitable a task as to hunt the flying shadows of an April day. In truth, it is hard to say what are the precise components of healthy urine. One chemist tells you one thing, another maintains the contrary, and who is to decide when none agree. A late writer on chemistry makes a few remarks upon this subject which are not undeserving of attention.

"It is difficult when so many substances exist together in a fluid to find out their state of combination; accordingly, very different accounts have been given of the saline ingredients of urine. During the evaporation of a fluid containing salts new affinities are brought into play, and compounds altogether different from those existing in it are procured. But another difficulty presents itself with regard to urine. The *urée*, during the evaporation, generates ammonia, and thus causes the decomposition of some of the compounds and the formation of others. Hence, instead of free uric and benzoic acids, urate and benzoate of ammonia are obtained; and in place of the super-phosphates, neutral-phosphates and ammoniaco-phosphates are deposited. It is evident from this, that the substances procured must vary materially, according to the mode of analysis."

Fortunately disagreements in minor points of chemical researches exert no prejudicial influence on practice, and, so far as we know, it matters little to surgeons whether the pyro-uric acid be or be not the succinic. The more prominent, more obvious, and therefore, in a practical view, the more important features are recognized and acknowledged on all quarters. We have said that the knowledge of the diseases of the urinary organs has latterly made perceptible progress in this country. Yet that advance has been rather by the few than by the many, by individuals of talent and acquirements, not by the general mass of the profession. Knowledge to be widely useful must be widely diffused, and in our profession it is of comparatively little benefit to mankind, if the chosen few are its sole possessors. Your rare gems in the cabinets of *virtuosi* are pretty to look at and nothing more. We hail then the publication of Mr. Brodie's experience on calculous diseases, with a high and unfeigned degree of pleasure. There are few men of such sterling practical attainments, very few indeed on whose opinions his professional brethren are inclined to place such complete reliance. He has reached the summit of his profession by no royal road, by diligent observation of the phenomena of disease, and unwearied cultivation of morbid anatomy, in youth, in manhood, and we trust in age. Those who know him best, and especially those practitioners whose opportunities of testing the value of his professional opinions have been most ample, will acknowledge the justice of the tribute we pay him. We are no sycophants; we do not "flatter to betray," but we had rather run the risk of being accused of flattery, than withhold a public expression of approbation, where approbation is deserved. We could not say less, and we will not say more. We proceed to the analysis, for an analysis it will be, of the lectures in question.

The urine in its natural state is composed of many ingredients, maintained in solution at the temperature of the body. Sometimes, although this temperature is unaltered, and the urine continues in the bladder or urinary passages, one or more of these ingredients is deposited in a solid form. The deposite may be in the form of small particles or sand, or of larger masses termed calculi; but whichever it be, the nature of the disease is essentially the same.

### I. Sand in the Urine.

The urine contains, as is well known, a large quantity of uric or lithic acid, not in a free state, for it is very insoluble, but usually in the form of lithate of ammonia, a very soluble salt, which causes the reddening of litmus paper by healthy urine. In very cold weather, the urine, as it cools, deposits the lithate of ammonia, blended with some animal matter. The lithate of ammonia also forms the principal part of the soft or uncrystallized sediment deposited in the vessel by the urine of persons who labour under dyspepsia, and some other diseases. If to healthy urine is added an acid which has a stronger affinity for ammonia than the lithic, such as lemon-juice, the lithate of ammonia is no longer precipitated, but, instead of it, pure lithic acid in the form of numerous small red crystals, resembling particles of cayenne pepper. This change may happen in the body also, from another acid in the urine, causing the precipitation of lithic acid sand even in the bladder. Dr. Prout says that this acid is the muriatic. Those who, from weak digestion, are liable to the formation of acid in the stomach, are also liable to the red sand. If the food be indigestible, or if taken in too large quantity, the same thing may happen in the most healthy person. The free use of fermented liquors, especially such as contain acid, or sugar which may become acid in the stomach, leads to the same result. Persons leading a sedentary life, who perspire little, are especially subject to the red sand. The perspiration, it is well known, is very acid, and it seems as if something was carried off by the skin in perspiration, which would otherwise cause the urine to be loaded with acid. Thus Sir Gilbert Blane long ago remarked that a disposition to calculous disorders is frequently combined with eruptions on the skin.

Urine containing a super-abundant acid, precipitating the red sand, is bright and transparent, and of a copper colour, like that of Madeira wine. The patient has generally dyspeptic symptoms, and frequently is liable to gout, between which and the formation of the red sand there is a close connexion. The same peculiar constitution, the same luxurious diet and inactive life dispose to both. The chalk-stones formed in the bursæ and cellular membrane of gouty persons are composed of lithate of soda. In the higher classes of society the deposition of red sand exists chiefly in adults, in the lower, among children. Adults in affluent circumstances lead a more luxurious and inactive life than their children; whilst, among the lower orders, the diet of children is frequently unwholesome, and the consequent disorders of their digestive organs neglected.

In many instances the red sand is voided without any particular symptoms, the patient accidentally perceiving it in the urine. In other cases, there are uneasy sensations in the loins, pain in the groins and in the course of the urethra, and sometimes discharge of a small quantity of blood from the urethra. When the latter is irritable and liable to spasmodic affections, the contact of the red sand induces spasm in it, a diminution of the stream, and even difficulty in voiding the urine. In such cases bougies will not cure the stricture, unless combined with the remedies which tend to prevent the formation of red sand.

It is of great importance to put a stop to the formation of red sand, for, in itself an evil, it may lead to the formation of a larger concretion in the bladder. It may almost invariably be prevented by the exhibition of alkaline medicines—potass, soda, lime-water, magnesia, ammonia. One or other may be preferable, according to circumstances, or it may be adviseable to give them in combination. If the lithic acid is deposited in small quantity, and the bowels too much relaxed, (which is rare,) lime-water may be useful. In weak persons we may give ammonia. Dr. Prout recommends the carbonate of potass in preference to that of soda, because under certain circumstances soda will combine with lithic acid, and form an insoluble salt as bad as itself, whilst the lithate of potass is perfectly soluble, and will pass off in the urine. On the whole magnesia is preferable to all, for, being in itself insoluble, it cannot enter the circulation, unless it has first become combined with acid in the stomach or intestine, and thence cannot pass out of the system so soon. The dose of the alkalies must vary, but from ten grains to two scruples of magnesia may be given daily; and the others in proportion. Whilst the carbonates agree better with the stomach than the pure alkalies, the carbonic acid does not interfere with their effects. Salts containing a mineral acid, as the sulphates, muriates, nitrates, are of no avail, but the tartrates and citrates produce the same results as the pure alkalies or their carbonates. Thus Sir G. Blane has recommended a very efficient way of exhibiting the carbonate of potass, namely, in a saline draught with excess of alkali.

A good deal of care is necessary in apportioning the dose in such individual case. If too little alkali is given, lithic acid is still deposited, though in smaller quantity; if too much, the urine is rendered alkaline, and the ammonio-magnesian phosphate thrown down. If magnesia is taken in too large quantity, the patient is liable to the formation of magnesian calculi in the intestines, composed of the magnesia mechanically blended with the fæces and intestinal mucus. They are not uncommon now when magnesia is carelessly taken and given. Mr. Wilson found many pounds of magnesia collected in the colon of a patient, above a contracted part of the rectum. In exhibiting alkalies each case should be made the subject of a distinct experiment, and the patient should co-operate with the surgeon. As healthy urine turns blue litmus paper a little red, alkalies should not be given so as to destroy this property altogether, still less to render the urine alkaline. If blue litmus paper reddened by immersion in a weak acid, or in healthy urine, is turned blue by that under examination, the patient is in danger of suffering from a deposition of the phosphates, and the alkalies must be given in smaller quantities. The time when the urine is most acid and alkalies principally required is after dinner; they should be given in three or four hours afterwards, for if sooner they interfere with digestion. Sometimes it is better for the patient to take his medicine when he wakes accidentally in the middle of the night. In many cases only a single dose of magnesia at bed-time is required; in others it should be taken in the middle of the day also.

But it has been truly observed that the exhibition of alkalies is not striking at the root of the disease. When there is costiveness purgatives should be given, and they are useful when the bowels are not particularly torpid. Mercurial purgatives are on the whole to be preferred. A blue-pill may be given every night, with a senna-draught every fourth morning; or a calomel pill once or twice in the week at bed-time, and the draught on the following morning. When the disease is connected with gout, the patient may take twenty drops of the vin. colchici, twice or three times daily at first, afterwards a senna-draught with forty or fifty drops of the vin. colchici occasionally in the morning. But most, after all, is to be effected by diet. If the patient is a great eater he must moderate and simplify his food, and incline to a vegetable diet, avoiding, however, undressed, and especially acid or acescent vegetables. If he commits excesses in drinking, let him give up fermented liquors, or use them very sparingly; the French wines are injurious, especially champagne, but our own punch is worse than any.

If the patient has been in the habit of dining late in the evening, and going to bed soon after a hearty meal, he should alter his habits in this respect. If he has led a sedentary life, he should walk or ride daily, so as to induce perspiration. Those who take much exercise, may commit imprudences in diet, which they could not otherwise do with impunity. Copious perspiration may be produced most effectually by the use of the sulphur, fumigating, or hot-air bath. The latter is of great advantage to those who lead an inactive life and are subject to gout, as well as to those who have too large a proportion of lithic acid in the urine. The perspiration produced is highly acid, reddening the litmus paper even more readily than the urine does.

The red is not the only sand deposited by the urine. Sometimes it deposits distinct white particles, minute crystals of the triple phosphate. Here it is alkaline, turning reddened litmus paper blue, or, if very alkaline, yellow turmeric paper brown. According to Dr. Prout, the formation of this white sand takes place in the following manner. The urine ordinarily contains the phosphate of magnesia, a soluble salt, and held in solution. In certain states of disease the urea becomes decomposed in the kidneys, and ammonia is evolved, which combines with the phosphate of magnesia to form the triple salt; this being insoluble, is precipitated in the form of a white sand. Dr. P. also says that the same state of system which leads to the decomposition of the urea and evolution of ammonia, leads also to a more abundant formation of the phosphate of magnesia, whence arises the immense deposition of white sand.

The state of the system which leads to the establishment of alkaline urine and of white sand is very different from that which is attended with too acid urine, and with red sand. The latter occurs in those who are over-fed and over-stimulated—the former is the result of debility, the symptom of an asthenic state of system, observed in those who are exhausted by too severe mental or bodily exertions, or who have long been worn down by mental anxiety. In many instances a course of mercury renders the urine alkaline, in some persons a single dose of calomel will do so, apparently from the debilitating effect of this mineral. In a weak person the exhaustion produced by an active purgative will do the same. As might be anticipated, the too abundant exhibition of alkaline remedies will lead to the same result. Injuries of the spine were first observed by Mr. Brodie to produce alkaline urine in the year 1807, and since that time the observation has been confirmed by general experience. This is equally the case whatev e part of the spine is injured, whether the bladder be or be not paralytic. It continues even after the patient has recovered from all his other urgent symptoms. The same thing occurs where there is disease of the spine independent of mechanical injury. In a case of *ramollissement* of the lower half of the medulla, a true case of *tabes dorsalis*, produced by excessive venery, one symptom was a half-paralytic state of the muscles of the lower limbs, another was the highly alkaline condition of the urine. At the commencement of the paraplegia the urine was excessively acid, and it became alkaline as the paraplegiac symptoms advanced. This confirms a remark of Dr. Prout's, that alkaline urine is often preceded by a too abundant formation of lithic acid. In females labouring under aggravated hysterical affections the urine is frequently alkaline, and deposits the triple phosphate in abundance. The same persons are also liable to have red or lithic sand in the urine, and not unfrequently the two kinds alternate with each other. It is astonishing what a quantity, sometimes of one sometimes of the other, occasionally passes off in these cases.

Those persons who habitually secrete alkaline urine are generally pale and sallow; incapable of much bodily and mental exertion; complaining of lassitude and weariness. When this state of things has existed for some time their bowels become irregular, and they exhibit other marks of debility. These are the symptoms generally present, but in some few cases it is otherwise. Mr. Brodie has a gentleman at present under his care voiding alkaline urine although his health is good, and he suffers only from a costive state of bowels.

Besides the triple phosphate, another salt, the phosphate of lime, is frequently to be detected in the urine. A small quantity seems to be occasionally generated by a diseased kidney; but the greater proportion is derived from another source.

"Dr. Austin, physician to St. Bartholomew's Hospital, in the year 1791, published a Treatise on Stone in the Urinary Bladder, in which he states that 'the main result of his inquiries has been, that the stone is formed generally in very small parts, and often in no degree whatever, from the urine as secreted in the kidneys, but chiefly from mucus produced from the sides of the different cavities through which the urine passes.' The late Mr. Chevalier, in the second volume of the Medico-Chirurgical Transactions, published some observations which were intended to confirm Dr. Austin's hypothesis. These notions, however, attracted but little attention, even when first promulgated; nor is this to be at all wondered at, when we consider how much they are at variance with a multitude of well known facts. Nevertheless, they are not absolutely without foundation. Dr. Austin was in an error, inasmuch as he mistook the exception to the general rule, for the rule itself; but no further. It is true that calculous matter in by far the greatest number of instances is a deposit from the urine, but under certain circumstances it is generated by the mucous membrane of the bladder. How this happens was first distinctly explained by Dr. Prout.

I have described to you, in a former lecture, the phenomena which belong to chronic inflammation of the mucous membrane of the bladder. One of its effects is the secretion of a ropy adhesive mucus in a most abundant quantity. This mucus is highly alkaline, containing the carbonate of soda, which is a soluble salt; containing also the phosphate of lime, which is insoluble. The latter is frequently seen presenting the appearance of white streaks in the mucus. In some cases it is produced in still larger quantity, and it comes away, not in the form of white sand, but in that of small white irregularly-shaped masses, resembling fragments of mortar." 7.

This formation of the phosphate of lime may take place where there is no triple phosphate in the urine, and sometimes on testing you find the urine itself acid, though the mucus is alkaline. To succeed properly in this experiment, the urine and the mucus should be tested just as the latter has been deposited. If you wait some time longer putrefaction begins, ammonia is evolved, and the whole is rendered alkaline. The triple phosphate and phosphate of lime have different origins, and either may exist in the urine without the other. They frequently, however co-exist, and this combination is probably produced in one of the following ways. The primary disease may be a secretion of alkaline urine in the kidneys. This irritates the membranous surfaces with which it comes in contact, and, if it continues for a certain time, induces chronic inflammation of the mucous membrane of the kidneys and ureters, extending to the bladder, and giving rise to the formation of a large quantity of adhesive mucus, containing the phosphate of lime. Or, the chronic inflammation of the mucous membrane of the bladder may be the primary affection. This cannot last long without affecting the constitution; it excites low febrile disturbance attended with much debility, a state of system very liable to occasion a secretion of alkaline urine in the kidneys. In one or other of these ways the triple phosphate and the phosphate of lime become associated with each other, sometimes one and sometimes the other being the original malady.

The treatment of patients affected with the white sand is to be conducted on very different principles from that which we adopt to prevent the formation of the red. The deposition of the triple phosphate argues great general debility, and whatever increases that debility aggravates the disease. Purgatives must be used with great caution, mercurial purges must be carefully avoided. Alkalies must be carefully avoided also, though Mr. Brodie has frequently seen them given, with the very worst effects, by those who had a notion that alkalies were good for the gravel. It has been said that alkalies are useful where the digestion is bad, although the urine at the time is alkaline. Mr. B. has every now and then seen a case in which *small* doses of soda were given with advantage, but he is sure that such cases are exceptions to the rule. The principle which leads us to avoid alkalies, leads us to exhibit acids. The vegetable acids are very fit to be employed where they do not disagree with the stomach, and the patient may drink lemonade, or eat oranges or lemons in the necessary quantity. If the vegetable acids disagree with the stomach, as they frequently do, the mineral acids may be substituted for them: from five to ten minims of the muriatic acid, or from fifteen to forty of the diluted nitric being given three times daily. The dose of the acid must be regulated by a frequent examination of the urine with test papers. Tonics, as bark, quina, bitter infusions, sulphate of iron, muriated tincture of iron, are likely to be useful. The diet should be plain, rather generous, consisting of a due mixture of animal and vegetable food. Fermented liquors may be taken in moderate quantity, and, for the most part, the acid wines, as Hock and Moselle, are preferable to others. Dr. Prout has pointed out the good effects of opium, hyosciamus, and other narcotics. If opium does not interfere with digestion, and in these cases it generally does not, from half a grain to a grain may be given twice or three times daily. The patient must avoid all severe exercise of body or mind, and if possible he should be agreeably occupied by some light employment. Courses of mercury, nay, a single dose of it, are likely to be injurious, as is the case with antimony and other diaphoretics.

"Where the phosphate of lime is deposited in consequence of a ropy mucous secretion from the mucous membrane of the bladder, you are in the first instance to endeavour to remove the cause on which the secretion depends, namely, the chronic inflammation of the membrane. I must refer you here to the observations, which I made in one of my former lectures, briefly recapitulating, however, what I then said on the subject. Bleeding not only does not tend to diminish the inflammation, but is actually injurious. The first thing to be done is to discover the cause of the inflammation, and to remove it, if possible. It may depend on stricture of the urethra, and may be relieved immediately on the stricture being dilated with a bougie. It may depend on a partial retention of urine in the bladder, in consequence of an enlargement of the prostate gland. The bladder must then be emptied artificially by the introduction of a gum catheter, once, or twice, or three times daily. It is seldom advisable in these cases to keep the catheter constantly retained in the bladder, for then the catheter becomes in itself a source of irritation, keeping up the inflammation of the bladder, and adding to the cause, on which the deposition of the phosphate of lime depends. Perfect rest in the horizontal posture, opium, suppositories, and narcotics by the mouth, will be useful also. The exhibition of the decoction of the root of the *pareira brava* is, in many instances, productive of excellent effects. It has a remarkable influence over the secretion of the ropy alkaline mucus. Injections into the bladder of warm water, and even of a weak solution of nitric acid, are sometimes useful; but of the cases in which these last remedies are to be recommended, I shall speak to you more particularly in a future lecture.

Where these two diseases, namely, the secretion of the triple phosphate of ammonia and magnesia by the kidneys, and of the phosphate of lime by the bladder, are co-existent (and this is a very common occurrence,) you must combine the two modes of practice which I have just described with each other. They are quite compatible, and in fact there are very few of the remedies which are useful in the one case which are not also useful in the other." 8.

### II. RENAL CALCULI.

There are various kinds of renal calculi, differing from each other in chemical composition; some of frequent, and others of rare occurrence. The most common are those composed of *pure lithic acid;* they are generally of round or oval form, of light brown colour, and tolerably smooth on the surface. Next in order of frequency are the *oxalate of lime*, or *mulberry* calculi; of dark colour, usually of irregular figure, with a number of small prominences on the surface. Thirdly, the *triple phosphate of ammonia and magnesia* is sometimes deposited in the kidney, but, so far as Mr. Brodie knows, a renal calculus is never composed entirely of this salt. Where a calculus has been lodged in the kidney for a considerable time, the triple phosphate is found to constitute its external layer, but the nucleus is either lithic acid or oxalate of lime. Fourthly, phosphate of lime calculi are occasionally formed in a diseased kidney, probably not from the urine, but from the other secretions of the organ. Mr. Brodie has in his collection two kidneys entirely filled with calculi of this description.

Renal calculi, composed of lithic acid, chiefly occur in those who have led luxurious and indolent lives, and begin to form, in most instances, at about forty years of age. Many such persons are also liable to gout, the two affections sometimes alternating, at others running on together. Some individuals void a great number of these calculi in succession. We find them of various sizes, from that of a pin's head to that of a horse-bean. Oxalate of lime calculi are much more rare, and when the disposition to them exists they are not formed in such numbers as the lithic acid calculi. A patient may void one and never void another, or he may pass a second after a lapse of many years. In one instance Mr. Brodie discovered five or six in one kidney after death; which was occasioned by extensive suppuration of the organ, and complete disorganization of its glandular structure. In reference to Mr. Earle's opinion that the formation of renal calculi may frequently be traced to a local injury affecting the loins and kidney, Mr. Brodie observes :—

"First.—Where a disposition to form calculi exists, a mechanical injury may (I doubt not) determine the disease to one kidney rather than to the other; but this disposition is so manifestly connected with a peculiar state of the system, and peculiar habits of life, (especially in cases of lithic acid calculi,) that we seem to be scarcely justified in regarding it as arising altogether from the agency of a local cause.

Secondly.—It is not improbable that, in some cases in which a mechanical injury has preceded the formation of calculi in the kidney, the first effect of it has been to occasion disorganization of the glandular structure and abscess, and that the calculi generated under these circumstances have been composed of the phosphate of lime, derived, not from the natural secretion of the urine, but from the morbid secretions of the diseased part; and corresponding to the concretions of the same kind which are not unfrequently met with in other diseased textures." 66.

The following very accurate account of the symptoms produced by the presence of a renal calculus, and its descent along the ureter is deserving of attention.

"When a small calculus is formed in the kidney, it usually occasions some degree of pain in the corresponding loin, and the urine is observed to be tinged with blood, especially after any jolting exercise. These symptoms, however, are by no means constant, and it often happens that the patient has no suspicion of his labouring under the disease until the calculus begins to descend from the kidney into the bladder. Even in its passage along the ureter, if the calculus be very small it may be productive of little or no inconvenience. If, however, it be large enough to occupy the whole diameter of the ureter, or in any degree to stretch or distend it, there is considerable suffering. When the calculus first enters the ureter, there is usually pain, referred to the region of the kidney and the groin. The pain is often very severe, and in that case attended with sickness and vomiting, prostration of strength, cold extremities, a feeble pulse, and a pallid countenance; in short, the patient is in what is commonly called a state of collapse. These symptoms are followed by pain referred to the inside of the thighs and the testicle; and frequently the testicle is drawn upwards to the groin by a spasmodic contraction of the cremaster muscle: no relief is experienced until the calculus has escaped from the lower orifice of the ureter, and entered the bladder; but as soon as this has happened, the patient's tortures (for they truly deserve that appellation) are at an end. The time occupied by the passage of the calculus along the ureter varies in different cases, according to the dimensions and figure of the calculus, and the impulse which it receives from the current of urine behind it. Sometimes the calculus may reach the bladder almost immediately; at other times it may be lodged in the ureter for many hours, or even for two or three days. Where the passage of it is thus protracted, the parts, to which the pain is sympathetically referred, become tender to the touch, and the testicle not unfrequently is actually inflamed and swollen, the inflammation of it continuing for some time after the cause which produced it has ceased to operate." 66.

Amongst the higher and more luxurious classes of society, and it is usually in an individual likely to suffer from gout, we sometimes remark a somewhat similar train of symptoms, although the cause is wholly different. The patient complains of pain, at first in the region of the kidney, then extending downwards in the direction of the spermatic cord to the groin; afterwards there is a frequent desire to make water, the effort being attended with considerable, and sometimes violent suffering. The urine is scanty, of deep pink colour, depositing a pink sediment; when tested, unusually acid. If not relieved by art, such symptoms may continue for several days—if suitably treated, they may subside in a few hours. Cupping on the loins; colchicum, at first in the dose of a drachm and a half, followed by two doses of half a drachm at intervals of four hours; and a senna draught will probably complete the cure. Mr. B. imagines that there is gouty inflammation of the kidney.

In most cases, a kidney-calculus gets into the bladder soon after its first formation; but in some it remains for a considerable time in the kidney, and is dislodged at last by some accidental circumstance. This happened to an old gentleman who was overturned in a carriage with some ladies. On reaching home the bladder was much distended—he strained violently to make water, and at length a kidney calculus, having the form of one of the infundibula, was projected with much force into the chamber-pot. In other cases, a long impacted kidney-calculus is dislodged in consequence of some change which takes place spontaneously in the organ, independently of mechanical injury. Sometimes calculi detained in the kidney produce no inconvenience, indeed no symptoms whatever, being frequently found after death, when they had not been suspected during life. In other instances there is pain in the loins, and the urine is occasionally tinged with blood, especially after jolting exercise, as riding on horseback. Though these symptoms seldom fail to indicate calculus in the kidney, they may arise from other causes. Mr. Brodie has once seen them produced by fungus hæmatodes in the kidney. In other cases he has thought that bloody urine depended on a relaxed state of the vessels of the kidney, but no dissection was obtained to corroborate the suspicion.

Dissection of the bodies of those who die with calculi of the kidney throws great light upon the subject. In the early stages of the disease, small portions of calculous matter are found imbedded in the mamillary processes; afterwards the calculous concretion partly projects into the infundibulum; by and bye it escapes into the infundibulum and pelvis of the kidney altogether. Probably it is now propelled by the urine into the bladder. It may remain in the kidney, increase in size from fresh deposits of calculous matter, or even grow so large as to occupy the whole of the pelvis of the kidney, extend into the infundibula, assume the form of the parts in which it is lodged, and bear some resemblance to a piece of madrapore. In these cases, the outer layers are commonly composed of the triple phosphate, the nucleus being lithic acid or oxalate of lime, more frequently the former.

The ureter is seldom completety obstructed. When it is so, the urine necessarily becomes accumulated in the infundibula—these are dilated into large membranous cysts—the glandular structure is expanded, and in a great measure absorbed, so that in some cases the kidney is at last composed of a large membranous bag, consisting of a number of cells, communicating with each other and containing urine. Sometimes the kidney is wasted, the only remnant being a membranous substance, adhering to an irregularly-formed calculus. In such circumstances, the other kidney supplies the place of the affected one, and does double duty in consequence. The two changes in the state of the kidney alluded to belong to the same series of events. The kidney having been dilated to a mere membranous cyst, the urinary secretion ceases, the accumulated urine is absorbed, the cyst collapses and contracts, and at length it becomes a mere capsule, in which the calculus remains imbedded. An enlarged kidney forms a tumour to be felt readily in the abdomen of a thin person. The preceding statements will account for the occasional disappearance of such tumours.

A calculus lodged in the kidney not unfrequently induces ulceration and suppuration of that organ. The pus may escape with the urine, and pass into the bladder, when there is little or no constitutional disturbance, the symptoms being local, and rather directing attention to the bladder than the kidney. In a lady the symptoms were, a frequent desire to make water, which was voided only in small quantities—a cutting pain referred to the neck of the bladder—at first muco-purulent secretion, and afterwards true pus in the urine. Sometimes the abscess thus produced in the kidney has no exit into the ureter. There is a different order of symptoms.

"There is pain referred to one loin, extending to the groin, sometimes upwards towards the scapula, or forward across the abdomen; not in general aggravated by exercise. Not unfrequently there is an irritable state of the bladder, though this symptom does not exist in the same degree as where the pus escapes with the urine. The patient suffers from a sense of remarkable lassitude and depression, and he has occasional rigors; sooner or later he falls a victim to the malady, and the following symptoms mark his approaching dissolution. The pulse is small; there is a total incapability of mental as well as of bodily exertion, an utter listlessness and disregard of all external circumstances. The pulse becomes so feeble that it can be scarcely felt, and the extremities are cold. Sometimes there is sickness and vomiting, at other times a diarrhœa, which it is scarcely possible to check by the most powerful astringents. The urine in these cases is generally secreted in very large quantity. It is also albuminous, being rendered opaque by heat and by the addition of nitric acid. Albuminous urine is met with almost invariably where there is abscess of the kidney; but you are not to conclude that this alone is a certain sign of the existence of abscess. Chronic inflammation of the kidney may cause the urine to be albuminous, although there is no abscess; and an admixture of even a small quantity of blood with the urine will produce the same effect." 69.

In a few rare cases an abscess, connected with calculi of the kidney, presents and bursts in the loins. Some have spoken of an operation for the extraction of calculi from the kidneys. The proposal is absurd; but nephrotomy is very practicable where Nature, by the formation of an abscess, has pointed out the exact situation of the calculi. And now of the treatment of renal calculi.

A person is voiding a number of small lithic acid calculi in succession. Those which are formed cannot be dissolved, but, by attention, new calculi may be prevented from being generated. Purgatives and alkalies, the colchicum where there is a disposition to gout, attention to diet, and to modes of life, are the means to be employed. Little can be done in the way of prevention, where the calculus is of the mulberry kind; fortunately this is much less likely to occur than the lithic acid. The formation of the phosphate of lime calculus in the kidney always indicates disease of that organ, probably abscess. Whatever it be, we cannot be wrong in administering the mineral acids. When a calculus is passing from the kidney to the bladder, we can do but little; we may give opium in large quantity when the pain is unusually intense, the hip-bath and abundance of diluting drinks. Mr. Brodie has sometimes thought that the patient derived benefit from the exhibition of an active purgative.

If there is reason to suspect that a stone is lodged in the kidney, it is desirable that it should be made, if possible, to pass into the ureter, before it is so large as to be incapable of being conveyed along it into the bladder. Hard trotting on horseback generally produces bloody urine, which shews that the calculus is made to undergo some change of position, a favourable circumstance. Diluents and diuretics may be useful; so are cupping or leeches where there is considerable pain in the loins and neighbouring parts. Sometimes the application of a belladonna plaster is serviceable. Mr. Brodie thinks that setons and issues are seldom so, except where disease in the kidney, especially abscess, has been induced. In such a case they are often of marked benefit. Where abscess is confined in the kidney, and there are symptoms of depression and exhaustion, art can afford little or no assistance; the patient struggles against his fate in vain.

When a calculus is impacted in the ureter, it might be supposed that this tube would continue to be dilated above the obstruction, until it should give way. Morgagni quotes such a case from another author; but Mr. Brodie relates two cases which prove that it does not always happen. In one, no urine flowed, nor could any be drawn off by the catheter in the bladder, the patient became comatose, and died convulsed on the twelfth day after the commencement of the attack. On dissection, there was no urine in the bladder. In one kidney there were several calculi—in the other none. In the ureter of the latter, high up, a calculus was wedged in. In a case which occurred to Mr. Travers, both ureters were completely obstructed by calculi. As in the first case, there was an entire suppression of urine.

### CALCULI OF THE BLADDER.

Any foreign body retained in the bladder for a certain length of time is liable to have calculous matter deposited upon it; and a calculus being thus generated, increases in size more or less rapidly, according to the composition of the urine. The most ordinary nucleus of a calculus, is one which has descended from the kidney; but a hazel-nut, a piece of flower-stalk, any foreign substance, in short, accidentally introduced into the bladder, may give rise to it. In the museum of the hospital are several small calculi, obtained from the bladder of a female, chiefly composed of the phosphate of lime, which indicates disease of the mucous membrane of the bladder, and each having a small fine hair, running longitudinally through its centre. Mr. Brodie suspects these hairs to be of a similar character with those which are occasionally found in encysted tumors and in other diseased structures. Mr. Brodie attended a gentleman with calculus in the bladder, who finally died of disease in the kidney. Every now and then Mr. B. detected small hairs in his urine, which he had reason to believe had come from the kidney. There was no post-mortem examination in either patient.

In cases of diseased bladder, where the mucous membrane is affected with chronic inflammation, earthy matter, composed chiefly of the phosphate of lime, is formed in small masses resembling mortar; any one of which may be retained in the bladder, and is liable to have calculous matter deposited upon it. Calculi, we need scarcely say, differ in their composition; and, for the light which modern investigations have thrown upon the subject, we are indebted to Wollaston, Prout, Brande, and other scientific chemists, whose names are familiar to the philosophic world. For the description of the various calculi we may refer to Mr. Brodie's summary, or to any standard work upon chemistry; it is sufficient for us here to enumerate their names. The substances then which enter into the composition of calculi of the bladder are—lithic acid—oxalate of lime—the triple phosphate of ammonia and magnesia—the phosphate of lime—the fusible calculus, or mixture of the two latter—the lithate of ammonia—lithate of soda—cystic oxyde—carbonate of lime, very rare—xanthic oxyd—and lastly, fibrinous calculus. Mr. Brodie found the latter in the bladder of a patient after death, who, during life, had not been suspected of labouring under stone. The kidneys had the appearance described by Dr. Bright as existing, when the urine is albuminous, but the urine had not been examined.

"In some cases we find a calculus composed throughout of one of the substances, which have been described, nearly pure; but at other times we find these substances variously combined with each other. The best mode of examining a calculus is to have it sawn through the centre. We then find, that in some of the compound calculi, the different substances are disposed in layers, the lithic acid distinct from the oxalate of lime; the oxalate of lime distinct from the triple phosphate, and so on; while in others they are intimately blended together.

It is only when they are divided in the manner which I have mentioned, that we can learn the true history of the formation of calculi. As Mr. Brande long ago observed, the centre or nucleus is generally either lithic acid or oxalate of lime. In many cases the additions to the calculus are of the same chemical composition with the nucleus; in other cases, we find the lithic acid deposited on the outside of the oxalate of lime; and more rarely, the oxalate of lime is deposited on the surface of the lithic acid. The deposit of lithic acid, or oxalate of lime, may take place in the bladder where there is no evident disturbance of the general health. If the general health becomes affected, and the bodily powers of the patient are impaired, either from the irritation of the stone in the bladder, or from any other cause, the urine becomes alkaline, and, in consequence, the subsequent additions to the calculus are formed of the triple phosphate of ammonia and magnesia. When the calculus has existed for some time in the bladder, it frequently happens, and indeed it always happens sooner or later, that the mucous membrane becomes inflamed; adhesive, tenacious mucus is secreted, which contains phosphate of lime; and this, being blended with the triple phosphate, forms the fusible calculus. Calculi formed in the ducts of the prostate gland, as I shall explain to you hereafter, are composed of phosphate of lime, pure, or nearly so; whatever may be the condition of the bladder, it very rarely happens that you find a simple phosphate of lime calculus in it. The phosphate of lime may be deposited in small masses, as I have explained to you formerly, but this nucleus being exposed to the contact of the urine, and the health becoming impaired, as always is the case under these circumstances, the triple phosphate is added to the phosphate of lime, so as to constitute a fusible calculus.

For these latter observations I am indebted to Dr. Prout. He has also furnished us with a knowledge of the following most important and interesting facts in the history of calculous formations. The phosphates very rarely form the nucleus of a calculus; but being once deposited, they continue to be so, and are never followed by other depositions. The phosphates may succeed the lithic acid, or the oxalate of lime, but neither of these ever succeed the phosphates. If the external surface of a calculus is composed either of the lithic acid, or of the oxalate of lime, you may be certain that there are no phosphates in the interior; whereas, if there are the phosphates on the outside, the general rule, to which there are but few exceptions, is that some other substance lies underneath." 131.

Calculous disorders prevail differently in different classes of society, at different ages, and in different climates and districts. Among the lower classes children are much more subject to them than adults; among the higher classes it is the reverse. The explanation has been already given when speaking of the tendency to lithic acid sand; for lithic acid forms the nucleus of the greater number of calculi. Persons of middle age in all classes are least subject to stone; women suffer less frequently than men, owing partly no doubt to their more temperate mode of life, partly to their greater capacity and simplicity of urethra. Mr. Brodie adverts to Mr. Copland Hutchison's notions respecting the infrequency of calculous disorders amongst sea-faring men. Mr. B. has operated on two officers in the navy, in whom the symptoms came on during actual service. Besides this, we must consider that sailors consist almost entirely of individuals neither very young nor very old, the class least liable to stone. It is altogether difficult to obtain precise data in such an enquiry.

Calculous diseases also prevail in particular districts, for which it is difficult to offer a satisfactory explanation. Thus, in the same district, persons are not only more disposed to lithic acid stones, but also to those of oxalate of lime, and it is not easy to imagine how peculiarities of diet, or of mode of life, should occasion both.

Calculi, for the most part, lie loose in the bladder, but sometimes they are encysted. In one specimen of this kind preserved by Mr. Brodie, there is enlarged prostate gland, which no doubt gave rise to the cyst, by preventing the bladder from being completely emptied. Here the cysts were formed by protrusions of the mucous membrane through the interstices of the muscular coat. In another specimen possessed by Mr. B. the stone was imbedded in a cyst near the fundus of the bladder, but the cyst is formed by dilatation of both the muscular and mucous tunics. The calculus was not so closely embraced by the cyst but that it might occasionally slip out; and it should be mentioned that the severe symptoms of stone during life would come on suddenly every now and then, the patient in general suffering little or nothing. Adherent calculi are very infrequent. It is not very uncommon for a diseased bladder to be incrusted in some part with calculous matter, but that is a very different thing from an adherent stone. Occasionally coagulable lymph is effused from the inflamed mucous membrane of the bladder, which also secretes the adhesive mucus, containing the phosphate of lime; a portion of this, mixed probably with the triple phosphate from the urine, is deposited on the lymph, just as, after lithotomy, the same sort of deposition will sometimes take place in the wound. In many cases there is only a single stone in the bladder, in others, two or three, or, occasionally, even many more than these. When there are more than one they are more or less polished from attrition, and assume the shape of irregular polyhedra.

The symptoms produced by calculi in the bladder next engage Mr. Brodie's attention. Different individuals suffer in very different degrees; and even the same individual does so at different stages of the complaint. The symptoms vary; 1st, according to the size of the stone, its smoothness, roughness, figure—2dly, according to the quality of the urine, whether too acid or too alkaline, in either case too stimulating—3dly, according to the state of the bladder, for nothing aggravates the symptoms so much as inflammation of the mucous membrane of the bladder. The symptoms themselves need not detain us, so many excellent decriptions of their course and order being in every body's possession.

In some instances the disease may exist for many years, without the symptoms becoming severe, but in general they are progressive and reach their height in the course of two or three years.

"At first, the patient's general health is unaffected; but by-and-by the health begins to suffer, the urine becomes alkaline, and the triple phosphate is deposited on the original stone. Now all the symptoms are much aggravated. The alkaline urine is more stimulating to the bladder than healthy urine, and this is one cause of the patient's increased sufferings. Another is, that that state of the general health which causes the alkaline urine to be secreted by the kidney, is attended with an increased or morbid sensibility of the nervous system generally.

As the disease advances, the continued irritation kept up by the stone, induces inflammation of the mucous membrane of the bladder. There is now a still further augmentation of the patient's sufferings. The stone is rolling about in an inflamed bladder, and you know how the sensibility of every organ in the body is increased by inflammation. The existence of this state of things is indicated by the greater pain, and by the desire to make water being almost constant; by the urine coming away offensive to the smell, soon becoming putrid and ammoniacal, and depositing the usual thick tenacious mucus streaked with blood. This mucus, as I have already explained to you, leads to the formation of the fusible calculus; and all that I have now stated will lead you to understand that different kinds of calculus are attended with different degrees of suffering. A patient with a simple lithic acid calculus, suffers less than one with a calculus composed externally of the triple phosphate; and the latter less than a patient with a fusible calculus. The oxalate of lime or mulberry calculus, on the whole, occasions more distress than the lithic acid calculus; probably on account of the irregularities which so frequently exist on the surface of the former: but it occasions less distress than the calculi composed of the phosphates." 155.

Patients with enlarged prostate gland are particularly liable to stone in the bladder, in consequence of the organ being never perfectly emptied, and small kidney calculi or lithic sand being detained in it. Sometimes an enlarged prostate occasions a calculus in another manner; the mucous membrane of the bladder becomes inflamed, the mucus which is secreted deposits the phosphate of lime in small mortar-like masses, and each of these becomes the nucleus of a calculus. On examining the body, you find several calculi of irregular forms, small size, of white colour, and rough on the surface.

"Patients with diseased and enlarged prostate do not in general suffer more from the stone in the bladder than other individuals. Indeed I am inclined to believe that, on the whole, they suffer less; probably in consequence of the tumour of the prostate preventing the stone falling down on the neck of the bladder. I have, however, seen three cases, in each of which there was a stone in the bladder, complicated, not only with an enlarged, but ulcerated prostate; and the sufferings of these patients were greater than I had ever before witnessed in persons labouring under the same disease. They were, indeed, most horrible. In two of these cases, the surgeon who was in attendance (as I think) indiscreetly performed the operation of lithotomy. One of them died in about five minutes after the operation; the other became immediately comatose, and died in a few hours. The third patient was admitted into our hospital, under the late Mr. Ewbank. The symptoms were precisely similar to those which existed in the two other cases, and Mr. Ewbank, on the result of these cases being stated, very properly determined not to perform an operation, although the man had come into the hospital for the purpose. The poor fellow died in two or three days afterwards, and, on examining the body after death, we found a large stone and an ulcerated prostate, as had been anticipated." 135.

Calculi in the bladder frequently induce spasmodic stricture, and from the increased efforts to expel the urine, the muscular coat of the bladder always becomes thickened. Stone in the bladder admits, in the male sex, of nothing approaching to a natural cure. It may be a year, it may be ten, or even more, before dangerous symptoms arrive, but sooner or later the stone will prove the cause of death. The immediate cause of the patient's destruction is, in the great majority of instances, inflammation of the mucous membrane of the bladder. Chronic inflammation may exist long, and admit of recovery, but if it becomes acute, or even approaches to it, the situation of the patient becomes dangerous, nay, desperate. The inflammation extends up the ureter to the kidneys, and even their glandular structure becomes affected. In a patient of Mr. Keate's, who had laboured under stone for many years, the kidneys were converted into a substance resembling fungus hæmatodes, though not decidedly such. Sometimes abscesses form in the kidneys, at others there is a collection of muco-purulent fluid found after death in the pelvis and infundibula. Sometimes inflammation extends to the loose cellular membrane round the bladder, and putrid sloughy abscesses are formed in it.

In a very few cases the bladder ulcerates, and the stone escapes from its cavity. A remarkable case of this kind occurred to Sir Everard Home. There was a fistula in perineo, he operated for the stone, and the latter was found lying in an ulcerated cavity in the perineum. The man died, the bladder was very small and ulcerated, and the ulcer communicated with the ulcerated cavity in the perineum.

Mr. Brodie suspects that abscess in the cellular membrane of the pelvis, unconnected immediately with the bladder is not an uncommon occurrence in those cases in which the patient is allowed to linger on and die of the disease. In a patient who died in a very short time after the operation of lithotomy, a very large abscess was found in the pelvis, communicating with the bladder by an ulcerated opening on one side of its neck. In another case, there was an abscess, occupying nearly the whole pelvis, but not communicating with the bladder. The patients died so soon after the operation that it appears evident to Mr. Brodie, that the abscesses must have existed before its performance.

